# Mixed planting of subtropical Chinese fir in South China improves microbial carbon source metabolism and functional diversity through the accumulation of nutrients from soil aggregates

**DOI:** 10.3389/fmicb.2024.1404428

**Published:** 2024-07-22

**Authors:** Jiazhen Deng, Jingda Hu, Yongzhen Huang, Shengqiang Wang, Shaoming Ye

**Affiliations:** ^1^Guangxi Colleges and Universities Key Laboratory for Cultivation and Utilization of Subtropical Forest Plantation, Guangxi University, Nanning, China; ^2^Experimental Center of Tropical Forestry, Pingxiang, China; ^3^State Dongmen Forestry Farm of Guangxi Zhuang Autonomous Region, Chongzuo, China; ^4^Guangxi Key Laboratory of Forest Ecology and Conservation, College of Forestry, Guangxi University, Nanning, China

**Keywords:** Chinese fir, mixed plantation, soil aggregate, carbon metabolism activity, soil microbial functional diversity, Biolog Eco microplate

## Abstract

**Introduction:**

Soil microbial functional traits are key indicators of soil microbial ecological traits; however, how mixing patterns of Chinese fir and broadleaved trees drive soil microbial functional trait variation at the aggregate scale and how soil microbial functional traits are linked to soil fertility factors have largely not been determined.

**Methods:**

In this study, soil from the 0–20 cm depths in three Chinese fir plantations was collected, and the soil samples were separated into >2 mm (large macro-aggregate), 0.25–2 mm (macro-aggregate) and <0.25 mm (micro-aggregate) by complying with an optimal moisture sieving procedure. The metabolic activities, functional diversity and different carbon sources utilization characteristics of the soil microorganisms were determined by the Biolog Eco microplate method.

**Results:**

In all Chinese fir plantations, micro-aggregates (<0.25 mm) consistently exhibited the highest levels of microbial metabolic activity, a more uniform carbon source utilisation capacity, and the highest microbial diversity. Micro-aggregates also showed elevated levels of soil organic carbon (OC), total nitrogen (TN), total phosphorus (TP), and higher ratios of C/N and C/P compared to large macro-aggregates and macro-aggregates, indicating that micro-aggregates contain more resources available to soil microorganisms. Soil OC, TN, and TP content were enhanced by integration with *Michelia macclurei*, suggesting that this combination promotes relatively favourable soil conditions for microbial growth and multiplication. This, in turn, promotes microbial metabolic activity. Furthermore, redundancy and correlation analyses showed that soil OC, TN, and TP were identified as principal determinants of soil microbial functional properties in Chinese fir plantations.

**Discussion:**

In summary, mixed cultivation and aggregate size influenced microbial functional properties via soil nutrient alterations. Consequently, adopting a mixed cultivation approach of Chinese fir and broadleaved trees is advocated in the subtropical regions of Guangxi. Employing a diversity of tree species, including *M. macclurei*, is recommended for optimal soil quality preservation.

## 1 Introduction

With the development of national forest and grass resource construction, China contributed 25% of the global new forest area from 2012–2022, with 8.76 × 10^7^ ha of planted forests preserved (Yang et al., 2019), ranking first in the world. Guangxi, Fujian and other forestry departments vigorously promoted the planting of Chinese fir (*Cunninghamia lanceolata*). Because of their fast growth, excellent material quality, high yield, ease of reproduction and other characteristics, Chinese fir plantations (CFPs) have become the mainstay of plantation forests (Zhou L. et al., 2020) and play an indispensable role in timber production and improving the functioning of China’s forest ecosystems. However, problems such as low-quality forests, more pure forests, fewer mixed forests, and soil fertility degradation exist in the management process related to the planting areas of CFPs (Wu et al., 2017). To address the above issues, numerous conservation initiatives have been undertaken to address these issues, and the development of mixed forests is considered an important practice for the good development of planted forests. Previous studies have shown that, compared with pure CFPs, mixed CFPs have obvious advantages in improving the soil nutrient content, enhancing the carbon (C) sequestration potential, and improving soil microbial reactivity (Guo et al., 2023; He et al., 2023). Consequently, mixing Chinese fir and broadleaved tree species may be a feasible way to address the decline in the service function of pure forest ecosystems. However, how mixing regulates soil function is still poorly understood. In recent years, ecologists have found that it has become particularly important to use methods centered around functional microorganisms to assess and explain ecosystem processes; thus, new discoveries may be needed in exploring changes in soil fertility from the point of view of functional soil microbial properties (Escalas et al., 2019; Yang et al., 2022).

Soil microorganisms, as “stabilizers” of ecological equilibrium, continually influence soil formation and development during their life cycle, driving nutrient cycling and plant growth (Ling et al., 2017). The functional characteristics of soil microorganisms are the main indicators of soil microbial ecology (Miltner et al., 2012; Wang et al., 2020; Zhou Z. et al., 2020) and include functions such as the degradation of plant and animal residues, protection against pests and diseases, humus formation, and the mobilization of nutrients (Trivedi et al., 2019); these characteristics are helpful for clarifying the roles of microbial communities in different environments (Preston-Mafham et al., 2002). Therefore, to fully understand the fertility of soil in which CFPs are found, greater attention should be given to the functional characteristics of soil microorganisms. Notably, stand planting patterns can affect microbial functional characteristics (Philippot et al., 2023); for example, increased tree species diversity can improve soil organic carbon (OC) stocks and affect soil microbial community C utilization (Duan et al., 2023). Microbial community activities are affected by the quantity and quality of apoplastic inputs when stand species composition and structure change (Monkai et al., 2018; Wang and Ye, 2020). Soil chemical properties are important for influencing the functional properties of soil microorganisms (Yin et al., 2018; Bertini et al., 2021; Liu et al., 2023). Low nitrogen (N) deposition was shown to improve the microbial biomass and microbial diversity of soil in CFPs, while moderate or high N deposition was inhibitory (Yuan, 2013). Additionally, the soil C and N contents and C/N ratio are the main factors affecting microbial biomass C, and microbial (especially bacterial) biomass and specific enzyme activity are significantly correlated with soil OC (Duan et al., 2023). In addition, microbial biomass increased by 7.3% with the addition of phosphorus (P). Fungal biomass and microbial P also increased significantly. One of the key mechanisms for the positive response of soil microbial biomass is the increase in below-ground biomass of come plants by adding P (Huang et al., 2016; Chen and Xiao, 2022). Consequently, revealing the functional properties of soil microorganisms in response to forest plantation patterns and the soil environment is vital to exploring soil quality and maintaining soil ecology.

The biomass and community functional diversity of soil microorganisms generally reflect their functional characteristics and community status (Rogers and Tate Iii, 2001; Gao et al., 2022). Changes in the functional diversity of soil microorganisms reflect overall community dynamics and can be measured by examining the extent of microbial C utilization. Biolog Eco uses substrate-induced metabolic response patterns to measure the metabolic functional diversity of soil microbial communities and utilizes these patterns to characterize changes in soil microbial community functioning (Sofo et al., 2010a,b) in the evaluation of the succession of different vegetation types (Jiang et al., 2021) and different environmental impacts (Lin and Xue, 2020). There are advantages in changing soil bacterial community characteristics, but most of these studies have focused on the whole soil level, and studies on the aggregate scale have rarely been reported. Aggregates with different size distributions are the basic units of soil structure and are important for soil microorganisms. The aggregates are equivalent to a natural barrier, controlling the interaction between microorganisms, enzymes and their substrates, thus affecting microbial activities (Wang et al., 2021). Previous evidence has suggested that the microbial functional properties of macro-aggregates are more susceptible to soil nutrient changes (Liao et al., 2022). Because different aggregate sizes have different soil morphological characteristics, pore distributions and numbers and other soil physical and chemical properties (Almajmaie et al., 2017), there is large variation in the distribution of water and air within these aggregates, which directly affects the exchange of matter and energy between microorganisms and the environment within the aggregates (Han et al., 2021). Taken together, these findings indicate that aggregates are the main factor affecting soil microbial diversity, therefore, to further clarify the link between improved soil structure and microbial function, further studies on microbial functional properties at the aggregate scale are needed.

In recent years, the ecological stoichiometry of soil C, N and P, the soil microbial mass; and the structure of soil microbial communities in association with CFPs have been widely studied (Chen et al., 2019; Hu et al., 2020; Cao et al., 2021; Mao et al., 2021; Zhang et al., 2021; Song et al., 2022); however, the functional characterization of microorganisms in soil aggregates has rarely been reported. Therefore, this work takes the CFPs of three stand types as the research object and adopts Biolog Eco technology to dissect the differences in the metabolic functional diversity of the soil microbial communities. Moreover, the changes in the soil microbial community functional characteristics with respect to the soil OC, TN, and TP contents and their stoichiometric ratios were analyzed. This provides a scientific basis for the use of microorganisms to improve soil ecosystem function, maintain soil health and ensure sustainable soil development in CFPs. The hypotheses were as follows: (i) soil microbial metabolic activity, C source utilization capacity and functional diversity change with different stand types and aggregate sizes and (ii) changes in stand type and aggregate size affect soil OC, TN and TP content and their stoichiometric ratios, which in turn drive changes in the microbial functional properties of soil aggregate.

## 2 Materials and methods

### 2.1 Experimental site

**Figure 1:**
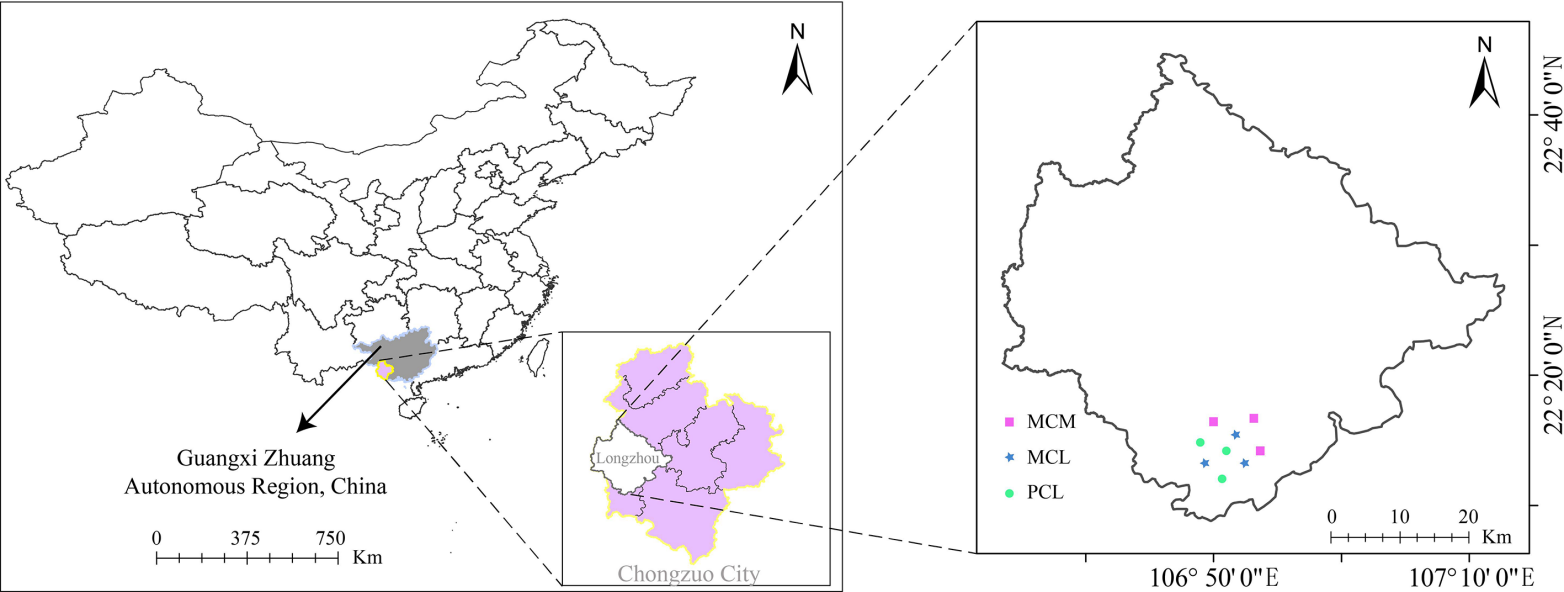
Location of the experimental site. MCM: Mixed plantation of *Cunninghamia lanceolata* and *Michelia macclurei*. MCL: *Mixed plantation* of *C. lanceolata* and *Mytilaria laosensis*. PCL: Pure plantation of *C. lanceolata*.

The sampling site was located at the Qingshan Experimental Field, Longzhou County, Chongzuo city, Guangxi (22°08′–22°44' N, 106°33′–107°12′ E) (Figure 1). This area has a southern subtropical monsoon climate, characterized by distinct dry and wet seasons, and the year can be divided into three seasons: cool, hot and rainy. The average annual temperature ranges from 20–22°C, with a maximum temperature of 43°C and a minimum temperature of −1.3°C. The average annual rainfall is 1,400 mm, and the average annual sunshine duration is 1,260 h. The landforms are mainly low hills, with an altitude of 500–900 m and a slope of 25–30°C. The parent rock driving soil formation is mainly granite, the soil type is mainly brick red soil, with moderate granularity, average water retention, and an acidic pH (pH 4.8–5.5), and the thickness of the soil layer is greater than 80 cm. The main shrubby vegetation in the understory consists of *Clerodendrum cyrtophyllum*, *Maesa japonica*, *Melastoma candidum*, and *Rubus alceaefolius*, which are used as understory shrubs in pure and mixed CFPs, while the herbs include *Miscanthus floridulus*, *Cyrtococcum patens*, *Pteris semipinnata*, and *Blechnopsis orientalis*.

### 2.2 Experimental design

In general, variation in soil space may confer some potential confounding effects. Therefore, similar landforms and representative CFPs were chosen to minimize these effects. In June and July 2019, after conducting field tests, on-site investigations and objective analysis on the *C. lanceolata* forest stands within the Tropical Forestry Experimental Center site, three representative stands with similar slopes and aspects and the same soil parent materials at the Qingshan Experimental Field, mixed stand of *C. lanceolata* and *Michelia macclurei* (MCM), mixed stand of *C. lanceolata* and *Mytilaria laosensis* (MCL), and pure stand of *C. lanceolata* (PCL) were selected for the experimental study based on topographic and geomorphic characteristics and ecological environment conditions. The three stands began to be planted in 1992, and their row spacing was 2 m × 3 m. The MCM stand was transformed from a pure forest through row thinning and has not been managed manually for many years. The MCL stand was created by inter-row mixture using a hole shaped land preparation method with a size of 40 cm × 40 cm × 30 cm on the pure Chinese fir plantation logging site. And it was weeded and tended during the growth process from 1992 to 1995. During the same afforestation period, the PCL stand was created in an adjacent area, with a density and spatial pattern similar to those of the mixed stands, with a survival rate of about 87%. A thinning was applied in 2008, reducing tree density by 26% (Li et al., 2021; Zhang et al., 2024a). The three stands have not experienced major natural disasters or other interference. The mixing ratio of the two mixed stands is 3:1. The general characteristics of the test site are shown in Table 1.

**Table 1:** Basic information on the sample plots in Chinese fir plantations with different stand types.

Stand type	Altitude (m)	Aspect	Slope (°)	Crown density	Litter quality (g m^−2^)	Average DBH (cm)			Average Height (m)		
						*Cunninghamia lanceolata*	*Michelia macclurei*	*Mytilaria laosensis*	*Cunninghamia lanceolata*	*Michelia macclurei*	*Mytilaria laosensis*
MCM	730	South	27°	0.85	723.66	17.55	24.48	—	14.05	15.96	—
MCL	725	South	23°	0.85	566.84	19.60	—	14.45	15.16	—	16.45
PCL	728	South	32°	0.85	340.58	21.42	—	—	16.53	—	—

MCM, mixed plantation of *Cunninghamia lanceolata* and *Michelia macclurei*; MCL, mixed plantation of *C. lanceolata* and *Mytilaria laosensis*; PCL, pure plantation of *C. lanceolata*. DBH indicates the diameter at breast height.

This study used a fully randomized design. Three replicates were used for each stand type, and 9 completely random typical squares (3 stands × 3 repeats) were obtained (*S* = 30 m × 30 m), with adjacent square spacings of approximately 1,000 m to avoid pseudoduplication caused by too close proximity (Figure 1). In each stand, *S* ≈ 10,000 m^2^. A sample square was randomly constructed >50 m from the stand edge to avert the impact of the forest margin effect.

### 2.3 Litter and soil sampling

Soil sampling in this study was performed at locations around the tree roots. Five sampling points were set up in an “S” shape within each sample plot. For each plot, 5 litterfall specimens were obtained from the soil surface in 5 random subplots (*S* = 1 m × 1 m), these were combined into a mixed litterfall specimen. In total, 9 (3 stand types × 3 replicates) mixed litterfall specimens were desiccated at 80°C until constant weight was obtained. And *in situ* samples were collected from the 0–10 cm and 10–20 cm soil horizons at each sampling point. After deep mixing, 18 mixed soil samples (3 stand types × 2 soil depths × 3 replicates) were obtained. The mixed soil samples were then stored in sterilized containers at 4°C for rapid transport to the laboratory. Under aseptic conditions, to avoid disrupting the aggregate structure, each mixed soil sample was gently broken down along its natural structure. The plant and animal debris and small stones were removed from the soil by passing through a 5 mm sieve.

### 2.4 Soil aggregate separation

Soil samples were separated into >2 mm (large macro-aggregates), 0.25–2 mm (macro-aggregates), and <0.25 mm (micro-aggregates) using the methods of Bach and Hofmockel (2014).

Specifically: the soil is placed in a sterilized container for drying at an ambient temperature of 4°C until the moisture content of the soil is about 10% before sieving; 300 g of the soil sample is then taken and placed on a 2 mm sterile sieve, where the sample is manually sieved for 4 min with a left-right amplitude of 5 cm and a frequency of 30 times per minute; the resultant soil less than 2 mm is further sieved with a 0.25 mm sterile sieve, and the sieving operation is repeated consecutively until there are enough soil aggregates with three different particle sizes, i.e., soil aggregates <0.25 mm, soil aggregates of 0.25–2 mm, and soil aggregates >2 mm. The aggregated soil is divided into two parts: one is placed in a cool and dry place in the laboratory to be completely air-dried for the determination of soil fertility factors of the soil aggregates, and the other is stored in a refrigerator at −20°C for the determination of microbial community functional characteristics of the soil aggregates.

### 2.5 Soil aggregates chemical analyses

The determination of soil OC was performed according to the methods of Nelson and Sommers (1996). TN was determined via the micro-Kjeldahl method (Bremner, 1996). The TP concentration was determined according to the color resistance of molybdenum and antimony (Bray and Kurtz, 1945).

### 2.6 Determination of the functional characteristics of the soil microbial communities

The soil microbial metabolic activity was determined by the Biolog Eco microplate method (Classen et al., 2003). Each microplate contained 96 wells that contained 3 replicates of 31 different C source substrates, as well as 3 controls. The 31 different C source substrates can be categorized into 6 classes: carbohydrates (10), amino acids (6), polymers (4), amines (2), carboxylic acids (7), and phenolic compounds (2).

Specific operations: 10 g of fresh soil is selected and placed in 90 mL of sterilized normal saline (0.145 mol L^−1^); after sealing, the sample is placed to a shaker for shaking at a frequency of 200 r min^−1^ for 30 min and then left static for 15 min; then, it is diluted by 500 times with sterilized saline, from which 150 μL of the supernatant is drawn with a pipette and inoculated into each well of an ecological plate; the plate is subsequently placed in a temperature constant incubator at 25°C for 14 days, during which the absorbance value is measured using a Thermo Scientific Microplate Reader (Thermo 1500, United States) every 24 h at a wavelength of 590 nm. The experimental operations are all to be completed on the ultra clean workbench, and the instruments and hands should be sterilized and disinfected before the experiment.

### 2.7 Statistical analysis

The average well color development (AWCD) per well is the mean value of absorbance of Biolog Eco plate wells, representing the average activity of microorganisms in the sample soil at different times, calculated with reference to Garland and Mills (1991). Based on the utilization of total C sources by soil bacteria in different treatments, the data of the 72nd h, when the AWCD value of C sources was stabilized and had a better fractal relationship between different treatments, were selected to calculate the functional diversity index (Kumar et al., 2017). The richness index characterizes the number of C sources utilized by microbial communities (Bell et al., 2005). The The Shannon-wiener index characterizes the richness of a species (Zak et al., 1994). The Simpson index characterizes species dominance (Wittebolle et al., 2009). The Pielou index reflects the number of C source utilization species and distinguishes the utilization degree (Shen et al., 2008). The formulas are:


(1) $$\mathrm{AWCD}=\sum \left(C_{i}-R_{i}\right)/N$$

(2) Richness=S

(3) $$H^{\prime }=-\sum \left(P_{i}\times \ln P_{i}\right)$$

(4) $$D=1-\sum {\left(P_{i}\right)}^{2}$$

(5) $$J=H^{\prime }/\ln S$$

In the above equations, the absorbance value of the *i*th C source hole is represented by *C_i_*, that of the control hole is represented by *R_i_*, and the total number of carbon sources is represented by *N* = 31. When the value of *C_i_* − *R_i_* in the C source hole is less than 0, it is recorded as 0, and the value of *C_i_* − *R_i_* is greater than or equal to 0. *S* is the total number of C sources utilized. *P_i_* is the proportion of the relative absorption of the *i* well to the total value of the relative absorption of all reaction wells.

All the data were analyzed using SPSS 26 (IBM, SPSS, NY, United States). The data were tested for normality using the Shapiro–Wilk test before ANOVA was performed. The effects of different stand types and aggregate sizes on the contents of OC, TN and TP; C/N (OC/TN), C/P (OC/TP) and N/P (TN/TP) were examined via one-way ANOVA, and the significance of these effects was examined via multiple comparisons via Duncan’s method. Redundancy analysis (RDA) and Monte Carlo permutation tests were performed using Canoco 5 (Canoco, NY, United States), and Pearson correlation analysis was performed and plotted using Origin 2021 Pro (Origin Lab, NY, United States).

## 3 Results

### 3.1 AWCD values of soil aggregate microorganisms

The AWCD values at both soil depths were greatest for the <0.25 mm aggregate size and decreased with increasing soil thickness. At the 0–10 cm soil depth, the AWCD values in the MCM and MCL stands were greater than that in the PCL stand. For the large maro-aggregates, significant differences in AWCD values were found between the MCL and PCL stands, while no significant differences were found between the MCM and PCL stands. For the 0.25–2 mm aggregates, the AWCD values of the MCM stands were significantly greater than that of the PCL stand. For the <0.25 mm aggregates, the AWCD values of the two mixed stands were significantly greater than that of the pure stand (Table 2).

**Table 2:** Effects of pure and mixed Chinese fir plantations on the ability of the soil aggregate microbial community to utilize total carbon sources.

Variables	Soil depth (cm)	Stand type	>2 mm	0.25–2 mm	<0.25 mm
AWCD	0–10	PCL	0.94 ± 0.05b	1.01 ± 0.09b	1.01 ± 0.02b
		MCL	1.13 ± 0.02a	1.20 ± 0.04ab	1.21 ± 0.07a
		MCM	0.99 ± 0.01b	1.25 ± 0.01a	1.25 ± 0.01a
	10–20	PCL	0.78 ± 0.03b	0.86 ± 0.04b	0.92 ± 0.03b
		MCL	0.84 ± 0.04ab	1.01 ± 0.04a	1.08 ± 0.05a
		MCM	0.95 ± 0.05a	1.03 ± 0.02a	1.09 ± 0.02a

MCM, mixed plantation of *Cunninghamia lanceolata* and *Michelia macclurei*; MCL, mixed plantation of *C. lanceolata* and *Mytilaria laosensis*; AWCD, average well color development; PCL, pure plantation of *C. lanceolata*. Data represent the average of three replicates ± standard error. The lowercase letters indicate significant differences among the same aggregate particle size among different Chinese fir plantations stand types (*p* < 0.05, *n* = 3).

At the 10–20 cm soil depth, the AWCD values were ranked as MCM > MCL > PCL. Regarding the >2 mm aggregate, the MCM stand had a significantly higher AWCD values than did the PCL stand; regarding the 0.25–2 mm and <0.25 mm aggregates, the MCM and MCL stands had a significantly higher AWCD values than did the PCL stand (Table 2).

### 3.2 Utilization of various C sources by microorganisms in soil

**Figure 2:**
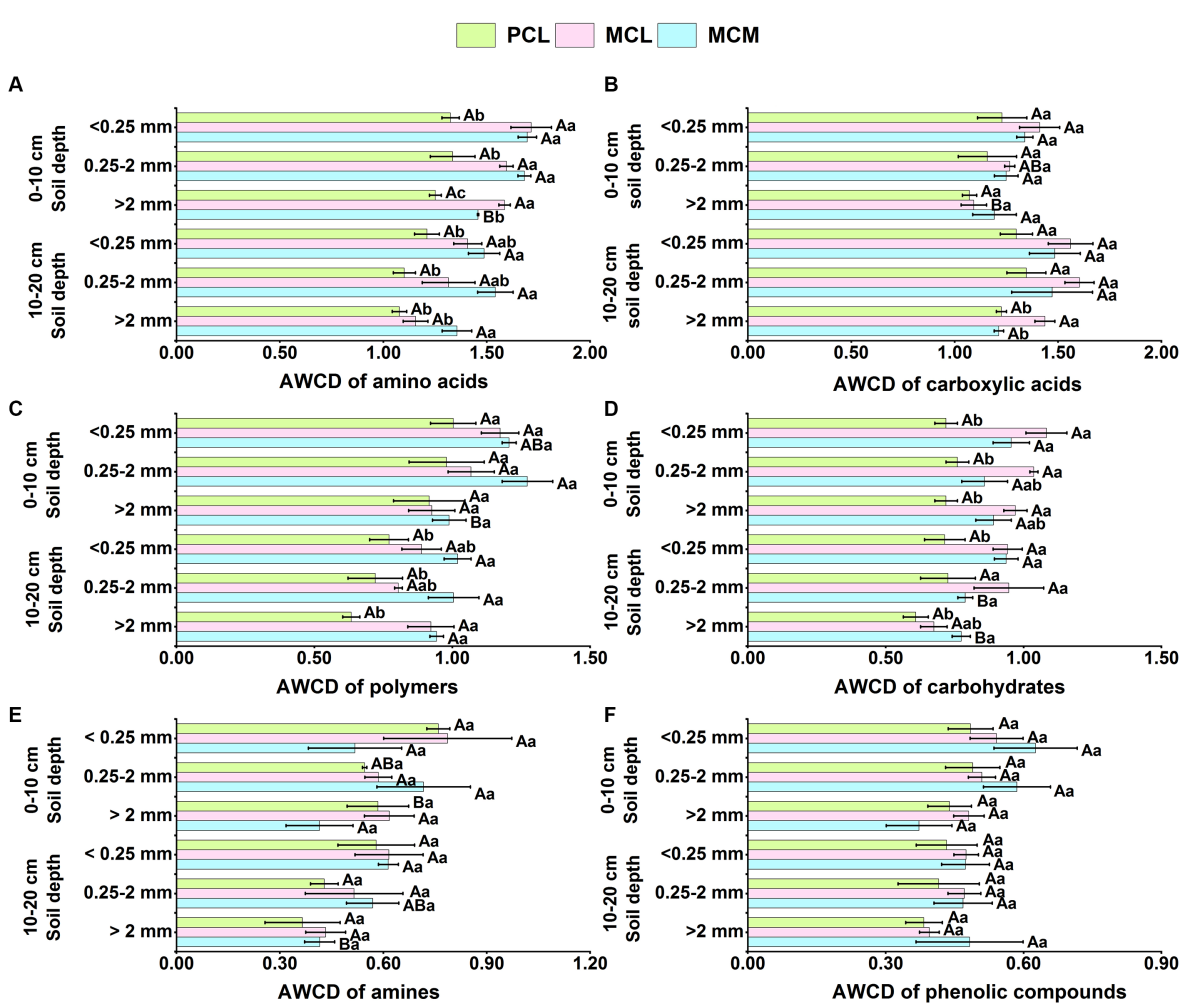
Effects of pure and mixed Chinese fir plantations on the ability of the soil aggregate microbial community to utilize various carbon sources. AWCD, average well color development. MCM, Mixed plantation of *Cunninghamia lanceolata* and *Michelia macclurei*. MCL, Mixed plantation of *C. lanceolata* and *Mytilaria laosensis*. PCL, Pure plantation of *C. lanceolata*. **(A-F)** Utilization of various carbon sources including amino acids **(A)**, carboxylic acids **(B)**, polymers **(C)**, carbohydrates **(D)**, amines **(E)** and phenolic compounds **(F)** by soil aggregate microorganisms within the soil depth of 0~20 cm in three Chinese fir plantations. Data represent the average of three replicates ± standard error. The lowercase letters indicate significant differences among different Chinese fir plantations stand types with the same aggregate particle size (*P* <0.05), and the capital letters indicate significant differences among the mean values of the three Chinese fir plantations stand types with different aggregate size (*P*<0.05).

As shown in Figure 2, the microorganisms of the three CFPs soil aggregates were more capable of utilising C sources from amino acids (1.08–1.71) and carboxylic acids (1.07–1.60), followed by polymers (0.63–1.36) and carbohydrates (0.61–1.04), whereas the utilisation of C sources from amines (0.37–0.79) and phenolic compounds (0.37–0.63) was at a relatively low levels (Figure 2).

At the 0–10 cm soil depth, the microbial community in the <0.25 mm soil aggregates in the MCM stand had a significantly greater capacity to utilize amino acids than that in the >2 mm aggregates (Figure 2A), and the microbial communities in the 0.25–2 mm soil aggregates had a significantly greater capacity to utilize polymers than did those in the >2 mm aggregate class (Figure 2C). In the MCL stand, the microbial community in the <0.25 mm aggregates was significantly more capable of utilizing carboxylic acid than those in the >2 mm aggregates (Figure 2B). The microbial community in the <0.25 mm soil aggregates in the PCL stand had a significantly greater capacity to use amines than did those in the >2 mm soil aggregates (Figure 2E).

At the 10–20 cm soil depth, the microbial community in the <0.25 mm soil aggregates in the MCL stand had the highest utilization capacity for carboxylic acids, significantly higher than that of the >2 mm aggregates (Figure 2B), and the microbial community in the <0.25 mm soil aggregates in the MCM stand had the highest utilization capacity for carbohydrates and amines, significantly higher than that in the >2 mm aggregates (Figures 2D,E). Under the three aggregate size conditions, the MCM stand contained significantly more amino acids and polymers than did the PCL stand (Figures 2A,C). The utilization capacity for carbohydrates of the microbial communities in the <0.25 mm and >2 mm soil aggregates in the MCM stand was significantly greater than that in the PCL stand (Figure 2D). The microbial community in the <0.25 mm aggregates in the PCL stand had a significantly greater capacity to utilize amines than did that in the >2 mm aggregates. Overall, the microbial communities in the soil aggregates in both mixed stands had greater capacities to utilize amino acids, carboxylic acids and carbohydrates than did those in pure stand. Among them, the microbial community of the MCL stand was significantly more able to use carboxylic acids and carbohydrate C sources than was that of the PCL stand.

### 3.3 Functional diversity of the soil aggregates

**Figure 3:**
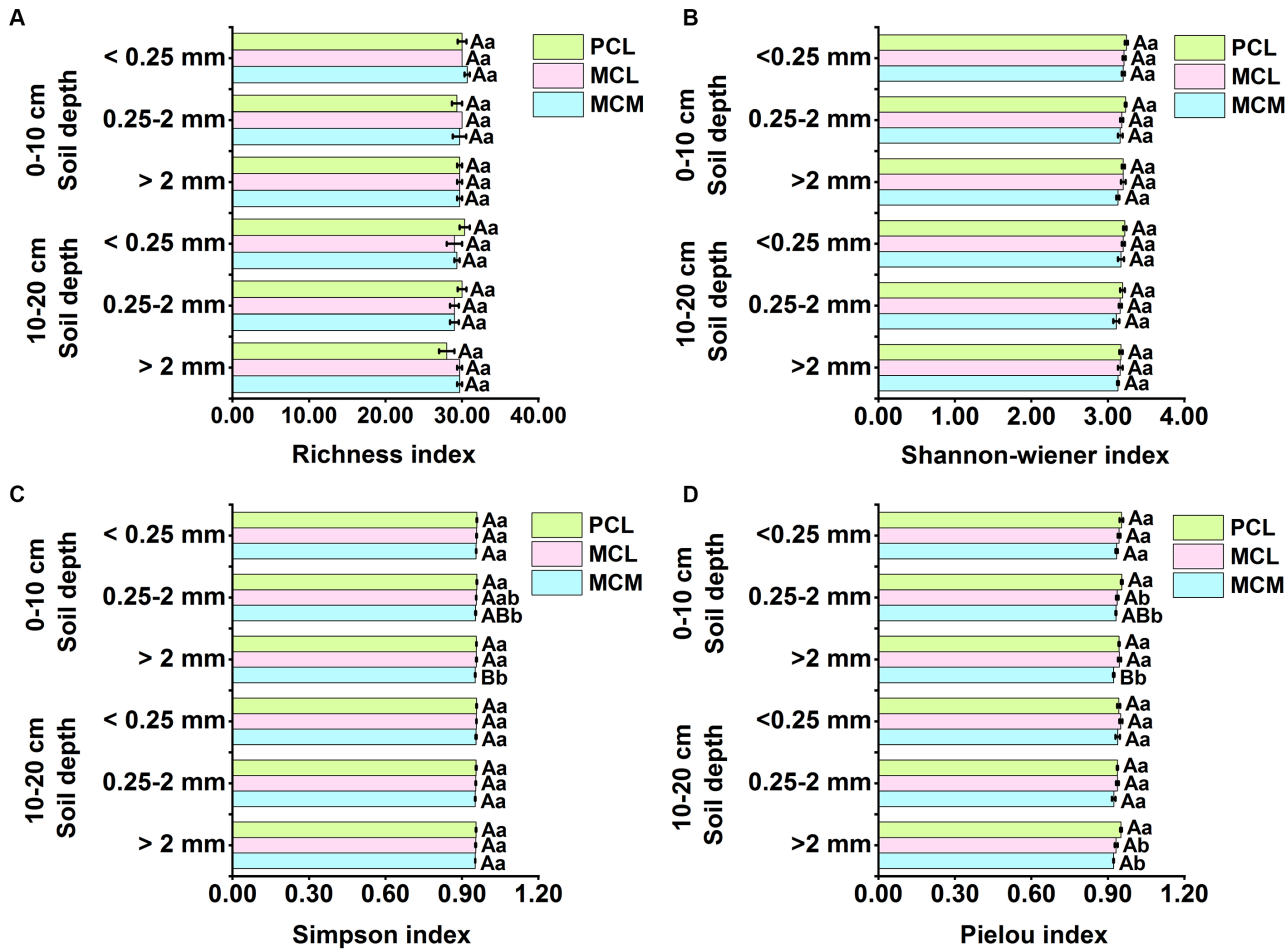
Effects of pure and mixed Chinese fir plantations on the functional diversity of microbial communities in soil aggregates. MCM, mixed plantation of *Cunninghamia lanceolata* and *Michelia macclurei*; MCL, mixed plantation of *C. lanceolata* and *Mytilaria laosensis*; PCL, pure plantation of *C. lanceolata*. **(A-D)** Richness index **(A)**, Shannon-wiener index **(B)**, Simpson index **(C)** and Pielou index **(D)** of soil aggregates microbial communities within the soil depth of 0~20 cm in three Chinese fir plantations, respectively. Data represent the average of three replicates ± standard error. The lowercase letters indicate significant differences among different Chinese fir plantations stand types with the same aggregate particle size (*P* < 0.05), and the capital letters indicate significant differences among the mean values of the three Chinese fir plantations stand types with different aggregate size (*P*<0.05).

At the 0–10 cm soil depth, the aggregate size did not have a significant effect on the richness index or the Shannon–Wiener index (Figures 3A,B). However, in the MCM stand, the Simpson and Pielou indices of the <0.25 mm aggregates were significantly greater than those of the >2 mm aggregates (*p* < 0.05) (Figures 3C,D). Stand type had a significant effect on the Simpson index and Pielou index, and for the 0.25–2 mm and >2 mm aggregate sizes, the Simpson index and Pielou index were significantly greater in the PCL stand than in the MCM stand, but there was no significant difference for the <0.25 mm aggregate size (Figures 3C,D).

At the 10–20 cm soil depth, aggregate size had no significant effect on the four functional diversity indices of the soil aggregate microbial communities of the three CFPs (Figure 3). Regarding the >2 mm aggregates, the Pielou index was significantly greater in the PCL stand than in the MCL stand (*p* < 0.05) (Figure 3D), while stand type had no significant effect on the richness, Shannon–Wiener or Simpson indices (Figure 3).

### 3.4 Soil OC, TN and TP contents and their stoichiometric ratios

**Figure 4:**
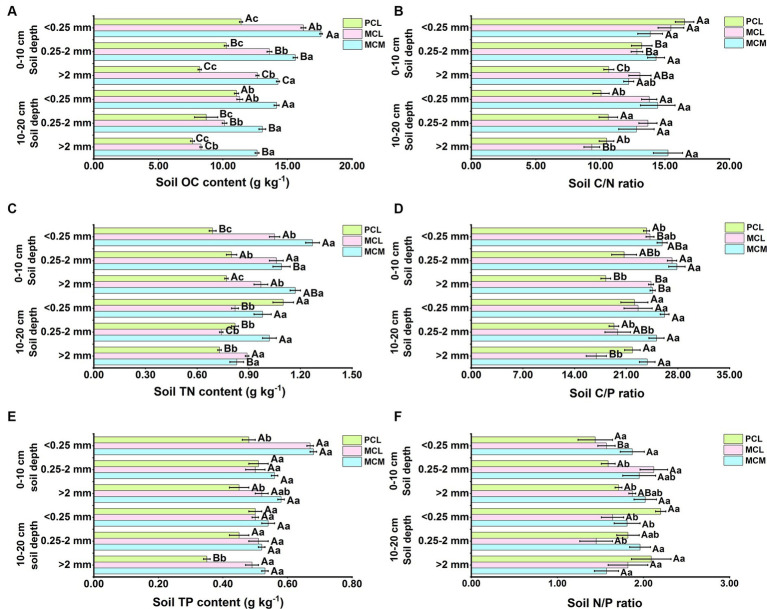
Soil organic carbon, total nitrogen, total phosphorus contents and the distribution of the soil C/N, C/P, and the N/P ratios as affected by stand type and aggregate size in Chinese fir plantations. MCM, mixed plantation of *Cunninghamia lanceolata* and *Michelia macclurei*; MCL, mixed plantation of *C. lanceolata* and *Mytilaria laosensis*; PCL, pure plantation of *C. lanceolata*. OC, TN, TP, C/N, C/P, and the N/P stand for organic carbon content, total nitrogen content, total phosphorus content, organic carbon/total nitrogen ratio, organic carbon/total phosphorus ratio, total nitrogen/ total phosphorus ratio, respectively. **(A-F)** OC content **(A)**, C/N ratio **(B)**, TP content **(C)**, C/P ratio **(D)**, TP content **(E)**, and N/P ratio **(F)** of soil aggregates within the soil depth of 0~20 cm in three Chinese fir plantations, respectively. Data represent the average of three replicates ± standard error. The lowercase letters indicate significant differences among different Chinese fir plantations stand types with the same aggregate particle size (*P*<0.05), and the capital letters indicate significant differences among the mean values of the three Chinese fir plantations stand types with different aggregate size (*P*<0.05).

Stand type and aggregate size significantly affected the soil OC, TN and TP contents and stoichiometry of the three CFPs (*p* < 0.05) (Figure 4). At the 0–10 cm soil depth, the soil OC content of all three stands decreased with increasing aggregate size, and the difference was significant (Figure 4A). The soil aggregate OC content in the MCM stand was highest for the micro-aggregates (Figure 4A), whereas the effect of aggregate size on the TP content in the three stands was not significant (Figure 4E). For the same aggregate size, the OC, TN, TP, C/P, and N/P ratios of the soil aggregates in the MCM stand were significantly greater than that in the pure stand (*p* < 0.05), and the OC and TN contents were significantly greater than that in the MCL stand (Figures 4A,C,D). At >2 mm aggregate sizes, the C/N ratio was greater in the mixed stands than in the PCL stand (Figure 4B). The C/P ratio was significantly greater in the MCM stand than in the PCL stand for all three aggregate sizes.

At the 10–20 cm soil depth, the soil OC and TP contents of all three stands were highest in the <0.25 mm aggregates (Figures 4A,E). Similarly, the C/N and C/P ratios of the soil aggregates in the MCL stand decreased with increasing aggregate size, and these differences were significant (Figures 4B,D). In contrast, the C/N, C/P, and N/P ratios in the MCM and PCL stands did not significantly differ among the three aggregate sizes (Figures 4B,D,F). Overall, the soil aggregate OC and TP contents as well as the C/N and C/P ratios were greater in the MCM stand than in the MCL and PCL stands for the same aggregate size. Overall, the contents of soil OC, TN and TP as well as C/N, C/P and N/P increased accordingly after mixing, and the effect of mixing was greater in MCM than in MCL stands.

### 3.5 Relationships between functional characteristics of the microbial community and OC, TN and TP contents and between functional characteristics and ecological stoichiometry

**Figure 5:**
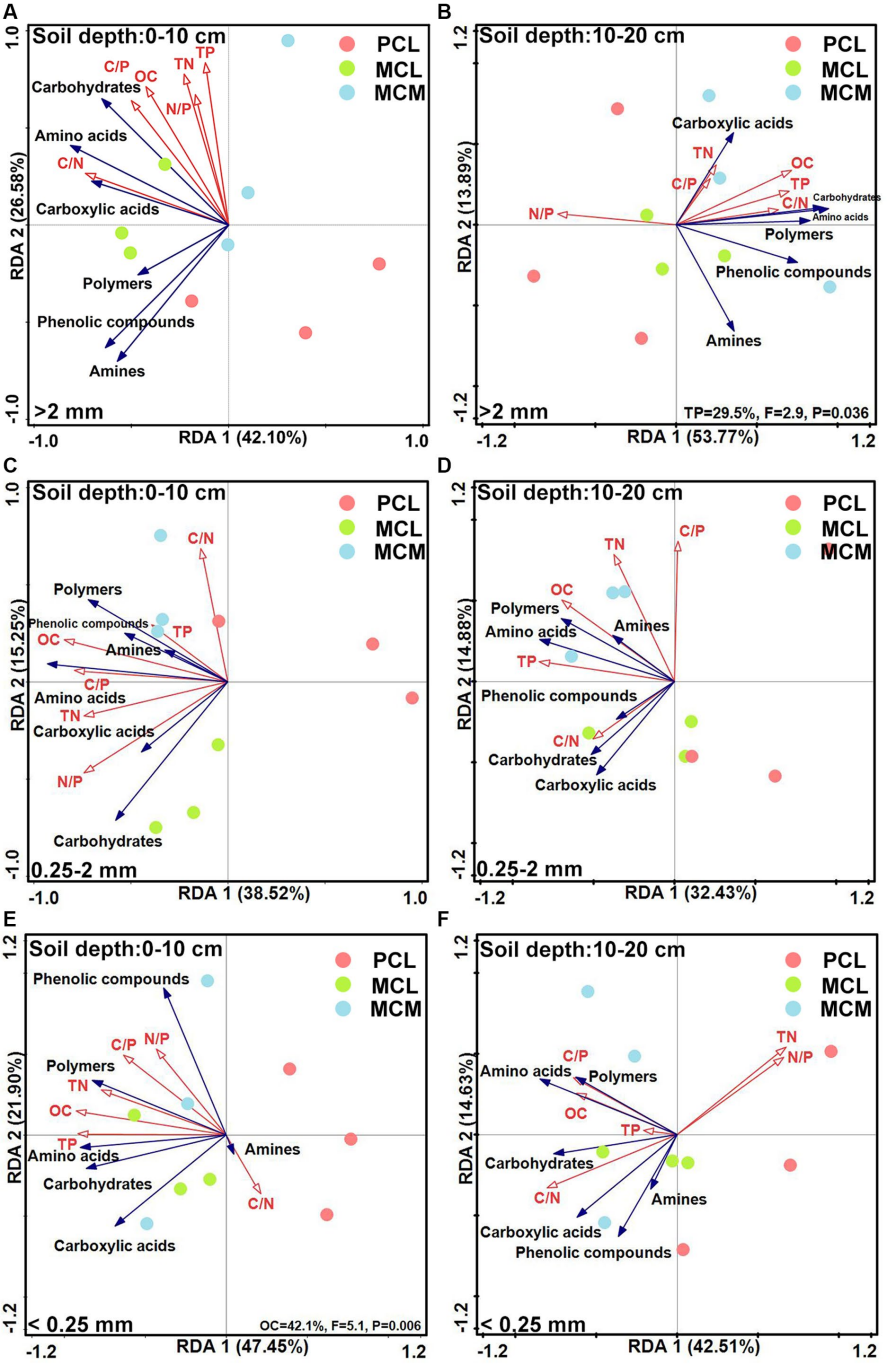
Redundancy analysis of the soil microbial community source utilization capacity and soil fertility factors across the three Chinese fir plantations with different aggregate fractions. MCM, mixed plantation of *Cunninghamia lanceolata* and *Michelia macclurei*; MCL, mixed plantation of *C. lanceolata* and *Mytilaria laosensis*; PCL, pure plantation of *C. lanceolata*. OC, TN, TP, C/N, C/P, and the N/P stand for organic carbon content, total nitrogen content, total phosphorus content, organic carbon/total nitrogen ratio, organic carbon/total phosphorus ratio, total nitrogen/ total phosphorus ratio, respectively. A --F: Redundancy analysis of soil aggregate microbial communities utilizing various carbon sources including amino acids, carboxylic acids, polymers, carbohydrates, amines and phenolic compounds compared with soil OC content, C/N ratio, TN content, C/P ratio, TP content and N/P ratio for different soil conditions, including 0~10 cm soil depth with >2 mm aggregates **(A)**, 10~20 cm soil depth with >2 mm aggregates **(B)**, 0~10 cm soil depth with 0.25~2 mm aggregates **(C)**, 10~20 cm soil depth with 0.25~2 mm aggregates **(D)**, 0~10 cm soil depth with <0.25 mm aggregates**(E)** and 10~20 cm soil depth with <0.25 mm aggregates **(F)**, respectively.

The RDA ranking diagram of soil aggregate microorganisms according to C source capacity, OC, TN and TP contents and stoichiometric ratio is shown in Figure 5. At the 0–10 cm in depth, the cumulative variance contributions of axis 1 and axis 2 for >2 mm, 0.25–2 mm and <0.25 mm were 68.68, 53.77 and 69.35%, respectively (Figures 5A,C,E). Regarding the >2 mm and 0.25–2 mm aggregate sizes, environmental factors had no significant effects on the ability of the soil microbial communities to utilize various C sources. Similarly, the degree of interpretation was 42.1% for <0.25 mm aggregates (Figure 5E).

At the 10–20 cm soil depths, the cumulative variance contributions of axes 1 and 2 for >2 mm, 0.25–2 mm and <0.25 mm were 67.66, 47.31 and 57.14%, respectively (Figures 5B,D,F). Among them, at >2 mm, TP had a significant influence on the capacity of soil microbial communities to utilize various C sources, with an explanatory degree of 29.5% (Figure 5B); at 0.25–2 mm and <0.25 mm aggregates, the OC, TN, and TP contents and their stoichiometric ratios had no significant effect on the C source capacity of the soil aggregate microbial community (Figures 5D,F).

**Figure 6:**
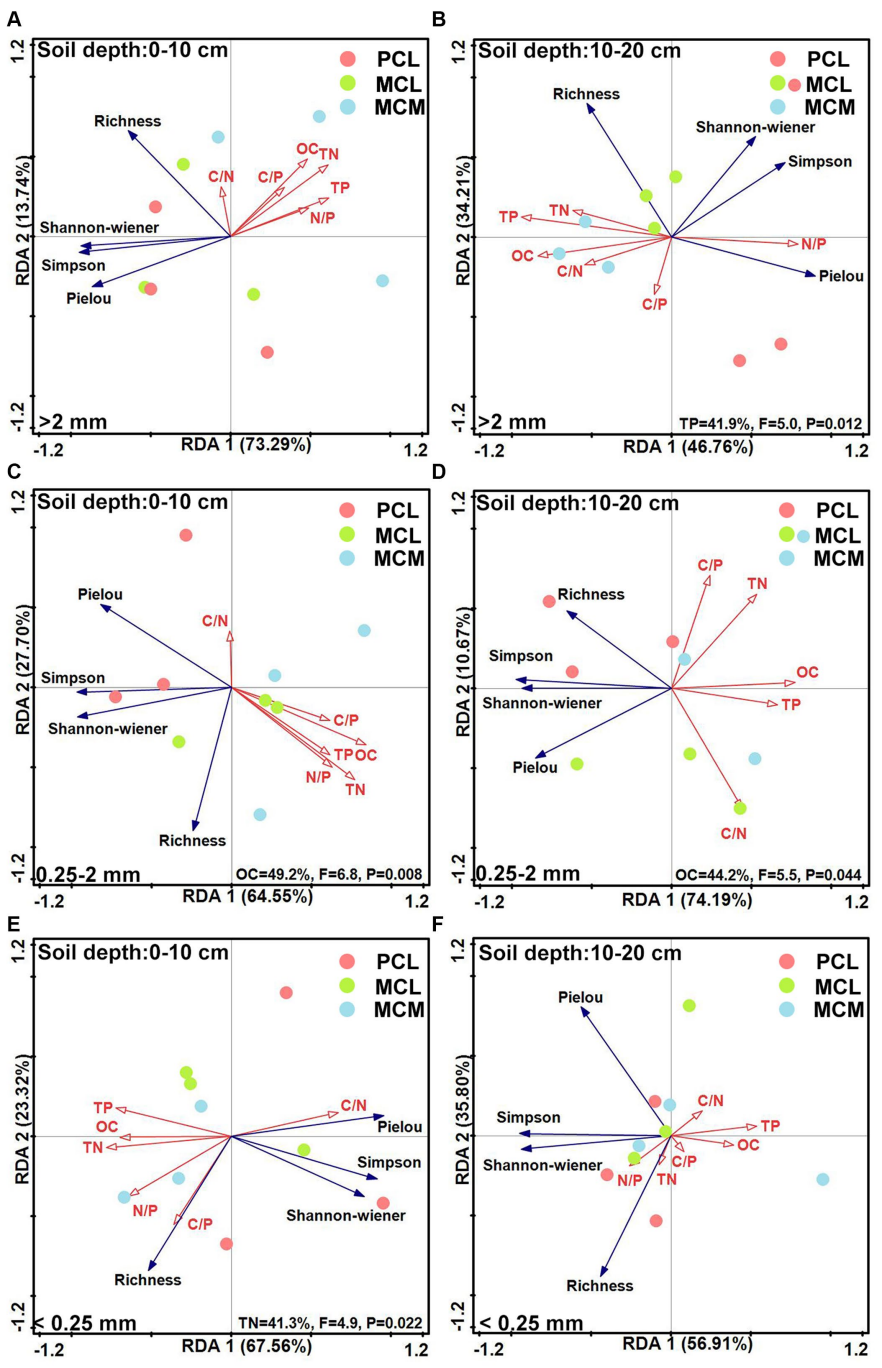
Redundancy analysis of the soil microbial community functional diversity indices and soil fertility factors across the three Chinese fir plantations within different aggregate fractions. MCM, mixed plantation of *Cunninghamia lanceolata* and *Michelia macclurei*; MCL, mixed plantation of *C. lanceolata* and *Mytilaria laosensis*; PCL, pure plantation of *C. lanceolata*. OC, TN, TP, C/N, C/P, and the N/P stand for organic carbon content, total nitrogen content, total phosphorus content, organic carbon/total nitrogen ratio, organic carbon/total phosphorus ratio, total nitrogen/ total phosphorus ratio, respectively. A --F: Redundancy analysis of Richness index, Shannon-wiener index, Simpson index, and Pielou index of soil aggregate microbial communities compared with soil OC content, C/N ratio, TN content, C/P ratio, TP content and N/P ratio for different soil conditions, including 0~10 cm soil depth with >2 mm aggregates **(A)**, 10~20 cm soil depth with >2 mm aggregates **(B)**, 0~10 cm soil depth with 0.25~2 mm aggregates **(C)**, 10~20 cm soil depth with 0.25~2 mm aggregates **(D)**, 0~10 cm soil depth with <0.25 mm aggregates **(E)** and 10~20 cm soil. depth with <0.25 mm aggregates **(F)**.

The functional diversity indices of the microbial community and the soil OC, TN and TP contents and their stoichiometric ratios according to the RDA model revealed that the OC, TN and TP contents had significant effects on the functional diversity of the microbial community. At the 0–10 cm soil depth, the cumulative variance contribution rates for >2 mm, 0.25–2 mm and <0.25 mm were 87.03, 92.25 and 90.88%, respectively (Figures 6A,C,E). Among them, in the 0.25–2 mm aggregates, soil OC content had the greatest effect of 49.20% (Figure 6C); in the <0.25 mm aggregates, the effect of soil TN content was significant, with a value of 41.30% (Figure 6E).

At the 10–20 cm soil depth, the cumulative variance contributions of axes 1 and 2 for >2 mm, 0.25–2 mm and <0.25 mm were 80.97, 84.86 and 92.71%, respectively (Figures 6B,D,F). Among them, in the >2 mm aggregates, TP had a significant effect, accounting for 41.9% of the total (Figure 6B); in the 0.25–2 mm aggregate size, OC had a significant effect of 44.20% of the total (Figure 6D). For aggregates <0.25 mm in size, the OC, TN, and TP contents and stoichiometric ratio had no significant effect on the ability of the soil aggregate microbial community to use C sources (Figure 6F).

**Figure 7:**
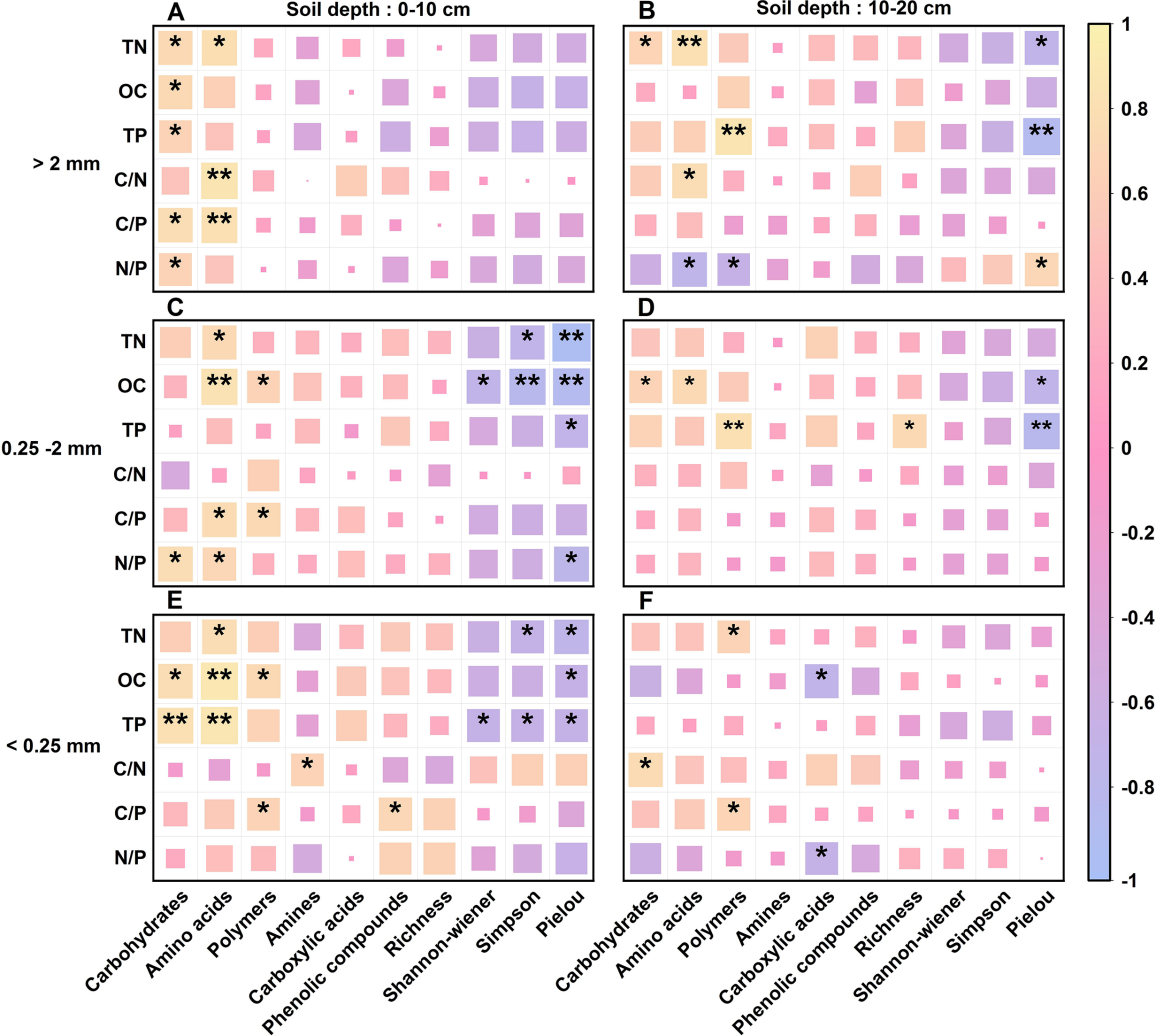
Correlation analysis of the soil microbial community function and soil fertility factors across the three Chinese fir plantations within different aggregate fractions. OC, TN, TP, C/N, C/P, and the N/P stand for organic carbon content, total nitrogen content, total phosphorus content, organic carbon/total nitrogen ratio, organic carbon/total phosphorus ratio, total nitrogen/ total phosphorus ratio, respectively. A—F: Correlation analysis of soil aggregate microbial communities’ diversity indexes (Richness index, Shannon-wiener index, Simpson index and Pielou index), their utilization of various carbon sources (amino acids, carboxylic acids, polymers, carbohydrates, amines and phenolic compounds) with soil OC content, C/N ratio, TN content, C/P ratio, TP content and N/P ratio for different soil conditions, including 0~10 cm soil depth with >2 mm aggregates **(A)**, 10~20 cm soil depth with >2 mm aggregates **(B)**, 0~10 cm soil depth with 0.25~2 mm aggregates **(C)**, 10~20 cm soil depth with 0.25~2 mm aggregates **(D)**, 0~10 cm soil depth with <0.25 mm aggregates **(E)** and 10~20 cm soil depth with <0.25 mm aggregates **(F)**. * indicates that the correlation is significant at the 0.05 level, and * * is significant at the 0.01 level.

Pearson correlation analysis revealed that OC, TN, TP, C/N, C/P, and N/P were positively correlated with the C source utilization capacity of the soil aggregate microbial communities of the different stand types (Figure 7). However, there were differences in the soil aggregates at the different soil depths, among which the effects of OC, TN, TP, C/N and C/P were significant.

At the 0–10 cm soil depth, the soil TP content and microbial ability to utilize carbohydrates were strongly significantly and positively correlated, while the OC and TP contents and the C/N and C/P ratios were highly significantly and positively correlated with the ability to utilize amino acids; however, the TN and OC contents were highly significantly and negatively correlated with the Simpson and Pielou indices of the microbial community. At the 10–20 cm soil depth, TN highly significantly and positively affected the microbial utilization of amino acids; TP highly significantly and positively affected the microbial utilization of polymers but highly significantly and negatively affected the Pielou index of the microbial community. There was a significant negative correlation between N/P and the utilization of both amino acids and polymers by the microbial community in the soil aggregates at the 10–20 cm depth. The utilization of C sources by soil microorganisms and the functional diversity of the community at the 10–20 cm soil depth were less affected by OC, TN and TP contents and their stoichiometric ratios than were those at the 0–10 cm soil depth.

## 4 Discussion

### 4.1 Metabolic activity and C source utilization in soil aggregates of different stand types

Soil organic matter (SOM) can be decomposed by microorganisms, thus laying the foundation for nutrient transformation (Berendsen et al., 2012), and microbial activity influences the efficiency of SOM decomposition, a process commonly measured by AWCD. The number of microorganisms and their community structure can be characterized by the AWCD values, with larger values indicating greater distribution density and greater activity. The present study showed that at the 0–10 cm and 10–20 cm soil depths, the AWCD values were lowest for the >2 mm aggregates and significantly lower than those for the 0.25–2 mm aggregates and <0.25 mm aggregates (*p* < 0.05) (Table 2), indicating that the soil microbial community activity in the >2 mm aggregates was poor; this result may be related to the soil OC content (Mekuria, 2013). Wen et al. (2016) investigated the functional diversity of soil microbial communities and found that the soil OC content could affect not only the metabolic activity of soil microbes but also the functional diversity of soil microbial communities in tree, shrub and herbaceous vegetation communities. The soil OC content in this study area was highest in the <0.25 mm aggregates and lowest in the >2 mm aggregates (Figure 2), suggesting that changes in aggregates can affect soil OC storage, which may ultimately lead to changes in soil microbial C metabolism activity (Tian et al., 2022). The results of this study are similar to those of Han et al. (2021).

Amino acids and carboxylic acids are the mainstays of plant root secretions and are correlated with soil nutrient effectiveness (Staddon et al., 1997), and amino acids have a positive effect on soil microbial biomass (Chari and Taylor, 2022). In this study, the ranking of the microbial communities in the soil aggregates in the three stand types was amino acids>carboxylic acids > polymers > carbohydrates > amines > phenolic compounds (Figure 2). Amino acids and carboxylic acids were the main substances secreted by the CFPs roots during the growing process, and they were the main C sources utilized by the soil microorganisms in this study area. These results are similar to those of Zhou Z. et al. (2020). Studies have shown that micro-aggregate resources are beneficial for microbial growth (Xue et al., 2021). In this study, the <0.25 mm aggregate size had the most significant effect on microbial community C source utilization. From the viewpoint of microbial community composition, different soil aggregates have different microbial community compositions (Rabbi et al., 2016), and microorganisms adapt differently to various types of substrates, resulting in certain differences in the ability of microorganisms to utilize various types of C sources (Han et al., 2021).

Vegetation composition and type are key factors influencing soil microbial activity (Saleem et al., 2019). In this study, the AWCDs of the two kinds of mixed stands were greater than those of the pure stand, and the values of the microbial community in the soil aggregates utilising various types of C sources at two soil depths were greater in the mixed stands than in the pure stand (Table 2). These findings indicate that the C metabolism activity and overall utilization capacity of the microbial communities in the soil aggregates in mixed stands were greater than those in pure stand. This is due to the rich vegetation composition of mixed stand with thicker humus layers (Table 2), which have higher apomictic production and decomposition rates (Tang et al., 2014); thus, the microorganisms are able to absorb more C from mixed stand. The main components of the apoplastic litter in pure Chinese fir forests are Chinese fir branches and leaves, which are thick and leathery, contain high amounts of lignin and cellulose, cannot be fully decomposed by microorganisms and are not favorable for nutrient recycling (Lin et al., 2011); therefore, the amount of C utilized by the soil microorganisms in pure stand will be less than that utilized in mixed stands.

### 4.2 Functional diversity of the soil aggregates in the different stand types

The richness, Shannon–Wiener, Simpson, and Pielou indices reflect the functional diversity of the microbial community (Li et al., 2020). In this study, neither the richness nor the Shannon–Wiener index was significantly responsive to either stand type or aggregate size (Figures 3A,B), suggesting that changes in the number or species of C sources utilized by the microbial community in the soil aggregates in the three stands did not significantly vary. At the 0–10 cm soil depth, the aggregate size significantly affected the Simpson index (*p* < 0.05) (Figure 3C), suggesting that aggregate size can influence the dominance of soil microorganisms, with the highest value for the <0.25 mm aggregates, indicating that soil microbial diversity was low at <0.25 mm aggregates. The microenvironment of microaggregate enables microorganisms to exert different enzyme activities and conversion forces from OC, TN and TP compounds, which affect microbial composition and activity, and the aggregate components and pores are selective for microorganisms, resulting in differences in microbial functional diversity in artificial forests with different size aggregates (Ling et al., 2014; Yudina et al., 2022), which is also one of the explanations for the soil structure and intermicrobial associations.

At both soil depths, the Pielou index of the >2 mm aggregates was significantly lower in the MCM stand than in the PCL stand (Figure 3D), suggesting that the soil microbial community in the MCM stand, which had a relatively variable relative share of the number of components of each species and a more concentrated capacity to utilize the soil microbial C source, thereby increasing microbial activity. In contrast, the soil microbial Simpson’s index was significantly lower in MCM stand with >2 mm and 0.25–2 mm aggregates than in PCL stand at 0–10 cm soil depth (Figure 3C), suggesting that the balance of soil microbial flora is higher in the MCM stand, which is similar to the results of the study by Beugnon et al. (2021). This finding suggests that mixed CFPs has an active effect on soil microbial diversity. The topsoil of mixed stands is enriched with high levels of apoplastic and root secretions and has high decomposition and rooting activity; thus, returning the C content to the soil provides a rich source of available C for microorganisms (Hartmann and Six, 2023). This resulted in an increase in the number and species of microorganisms that were highly coordinated with the C source material in the Biolog Eco microplates, which in turn led to a significant increase in microbial diversity (Zhang et al., 2024b).

### 4.3 Soil OC, TN and TP contents of the soil aggregates in the different stand types

The soil OC, TN, and TP contents and stoichiometric ratios can reflect the influence of different vegetation types on soil nutrients to a certain extent (Wang and Yu, 2008; Berlinches de Gea et al., 2023). In this study, the OC, TN, and TP contents and the C/N, C/P and N/P ratios at the 0–10 cm and 10–20 cm soil depths indicated that soil nutrients were mainly distributed in <0.25 mm aggregates (Figure 4), which is consistent with the findings of Tang and Wang (2022). Since microaggregates are <0.25 mm (John et al., 2005) and because of the presence of clay minerals and soil microorganisms in microaggregates (Li et al., 2019), these factors promote the aggregation of soil OC, TN, and TP in micro-aggregates (Six and Paustian, 2014), so the OC, TN, and TP contents of soils with <0.25 mm aggregates were the highest. One study showed that the soil OC and TN contents increased after interplanting broadleaved trees under CFPs (Sun et al., 2021). In the present study, the soil OC content was significantly greater than that in pure stand, and the soil TN and TP contents were also greater than those in pure stand (Figures 4A,C,D), indicating that mixing can improve the soil OC, TN and TP contents.

The soil C/N ratio, a predictor of the SOM decomposition rate, is usually inversely related to the soil decomposition rate. In this study, the overall C/N ratio was greater in mixed forests than in pure Chinese fir forest at the 0–20 cm soil depth (Figure 4B), suggesting that SOM mineralization and decomposition are slow in mixed forests, which is conducive to organic matter accumulation. The C/P ratio is considered an indicator of the soil P mineralization ability, as well as the release potential and P uptake potential of microorganisms, while the N/P ratio reflects the restrictive characteristics of soil N and P (Khan et al., 2016). In this study, both the soil C/P and N/P ratios were significantly greater in the mixed stands than in the pure stand (Figures 4D,F), indicating that the mixed pattern positively affects the soil stoichiometric ratio, which contributes to the enhancement of soil microbial activity as well as the fixation of soil P (Spohn and Chodak, 2015). In this study, the N/P ratio of the 0–20 cm soils of the three CFPs was lower than that of the surface soils on Chinese land (9.3) (Tian et al., 2010), suggesting that the soils of the three stands may be N limited due to insufficient N inputs. Mixing resulted in distinct amounts of C, N, and P sequestered by Chinese fir from air and soil, with the best mixing occurring in the MCM stand is an excellent native broadleaved species, and after a mixed forest with Chinese fir is formed, the apoplastic material is more easily decomposed, and contributing factors such as aboveground biomass and humus increase, which directly impact the accumulation of soil OC and TN. Moreover, the increase in species diversity in mixed stands results in an increase in the total resource use efficiency of light and water, resulting in increased plant productivity and accumulation of soil nutrients in forests.

### 4.4 Relationships between the functional characteristics of microbial communities; OC, TN and TP contents; and the ecological stoichiometric ratios of soil aggregates in different stand types

The characteristics of the soil microbial community structure and diversity are usually influenced by soil physicochemical properties (Chen et al., 2021). For example, the OC content is usually correlated with microbial biomass and microbial activity (Miltner et al., 2012; Lange et al., 2015), while N can limit microbial biomass and reduce microbial community diversity, and P addition can increase microbial biomass (Zeng et al., 2021; Chen and Xiao, 2022). The redundancy analysis showed that the microorganisms in the aggregates at the 0–10 cm soil depth responded strongly to the C source capacity and functional diversity of OC, compared with TP at the 10–20 cm soil depth (Figures 5B,E), indicating that OC and TP were important predictors affecting the functional characteristics of the soil microorganisms at the 0–20 cm soil depth in the study area. Mixed plantations with broadleaved trees leads to increased investment in plant residues, providing more C input to soil microorganisms in this region (Huang et al., 2017); therefore, it is speculated that increased OC and TP contents due to mixing stimulate microbial number and activity, thereby promoting soil respiration (Fornara and Tilman, 2008; Lange et al., 2015; Chen et al., 2019), which in turn improves the effect of microorganisms on C source utilization, which also reflects the high primary productivity of mixed stands.

The correlation analysis showed that OC, TN, TP, C/N, C/P and N/P all had positive correlations with the utilization of C sources in the soil microbial communities of the different CFPs stand types. The soil OC and TP contents and the C/N and C/P ratios were highly significantly different (Figure 7), indicating that the dynamic balance of soil OC, TN and TP affects soil microbial metabolism and the utilization of C sources. However, N/P significantly limits the microbial uptake of amino acids and polymers (Figure 7), which is related to the imbalance of N and P inputs, which can cause changes in ecosystem structure and function, indicating that soil P may be an important predictor of local soil microbial growth. In this study, the utilization of C sources and the functional diversity of the microbial community in soil aggregates at the 0–10 cm soil depth were both more affected by the OC, TN and TP contents and their stoichiometric ratios than those at the 10–20 cm soil depth, suggesting that the soil nutrients in the CFPs were mostly concentrated at the 0–10 cm soil depth. This difference may be due to the greater plant C input and greater microbial activity at the 0–10 cm soil depth, where the microorganisms use more plant litter and root secretions as a carbon source than microorganisms at the 0–20 cm soil depth (Pries et al., 2018; Button et al., 2022; Kong et al., 2022).

## 5 Conclusion

The stand type significantly influenced the functional traits of the soil microorganisms. Compared with those in pure stand, the soil microbial metabolic activity, C source utilization capacity and soil OC, total TN and TP content in mixed stands were significantly greater, especially in the MCM stand. At the aggregate scale, the C metabolic activity of soil microorganisms increased with decreasing aggregate size. Micro-aggregates consistently exhibited high C source utilization capacity and store more soil OC, TN, and TP than large macro-aggregates, also had a higher diversity of soil microorganisms, and these results support the first hypothesis. Soil OC and TP were key drivers of the functional properties of soil microorganisms. Changes in the soil OC, TN and TP contents and the soil stoichiometry of CFPs can significantly change microbial functional characteristics and thus promote the soil nutrient cycle, and these results support the second hypothesis. In conclusion, we showed that mixed plantations and aggregate size drive microbial functional traits through their effects on the nutritional aspects of soil. This study further deepens the understanding of the changing microbial functional characteristics of soil aggregates in different stand types and provides new insights into maintaining ecological quality enhancement and soil health in CFPs. In order to gain more comprehensive understanding of soil microbial community characteristics in CFPs ecosystems, it is necessary to further study soil microbial structure using high-throughput sequencing methods at the aggregate scale. At the same time, the effect of the litter also needs to be considered.

## Data availability statement

The original contributions presented in the study are included in the article/supplementary material, further inquiries can be directed to the corresponding author.

## Author contributions

JD: Data curation, Formal analysis, Investigation, Visualization, Writing – original draft. JH: Data curation, Formal analysis, Investigation, Methodology, Writing – review & editing. YH: Data curation, Investigation, Methodology, Writing – review & editing. SW: Methodology, Visualization, Writing – review & editing. SY: Methodology, Writing – review & editing, Funding acquisition.
